# The Association of Contemporary Screen Behaviours with Physical Activity, Sedentary Behaviour and Sleep in Adolescents: a Cross-sectional Analysis of the Millennium Cohort Study

**DOI:** 10.1007/s12529-022-10077-7

**Published:** 2022-03-11

**Authors:** Elli Kontostoli, Andy P. Jones, Natalie Pearson, Louise Foley, Stuart J. H. Biddle, Andrew J. Atkin

**Affiliations:** 1 School of Health Sciences, Faculty of Medicine and Health Sciences, University of East Anglia, Norwich Research Park, Norwich, NR4 7TJ UK; 2 Norwich Epidemiology Centre, University of East Anglia, Norwich, UK; 3 Norwich Medical School, Faculty of Medicine and Health Sciences, University of East Anglia, Norwich, UK; 4 School of Sport, Exercise & Health Sciences, Loughborough University, Loughborough, UK; 5 Centre for Diet and Activity Research at the MRC Epidemiology Unit, University of Cambridge, Cambridge, UK; 6 Centre for Health Research, University of Southern Queensland, Springfield, Australia

**Keywords:** Cross-sectional, Adolescents, Screen behaviours, Sedentary behaviour, Physical activity, Sleep

## Abstract

**Background:**

Screen behaviours are highly prevalent in adolescents and may be adversely associated with physical and mental health. Understanding how screen behaviours inter-relate with physical activity and sleep may help to clarify pathways through which they impact health and potential routes to behaviour change. This cross-sectional study examines the association of contemporary screen behaviours with physical activity, sedentary behaviour and sleep in adolescents.

**Method:**

Data are from sweep 6 (2015/2016) of the Millennium Cohort Study, conducted when participants were aged 14 years. Outcome variables were accelerometer-assessed overall physical activity and moderate-to-vigorous physical activity (MVPA), self-reported sedentary behaviour and sleep duration. Screen behaviours were assessed using a 24-h time-use diary. Multivariable regression was used to examine the association between screen behaviours and each outcome variable separately for weekdays and weekend days.

**Results:**

The use of social network sites was associated with (beta coefficient, 95% confidence interval (CI); minutes/day) less time in MVPA (weekdays: − 5.2 (− 10.3, − 0.04); weekend: − 10.0 (− 15.5, − 4.5)), and sedentary behaviours (weekdays: − 19.8 (− 31.0, − 8.6); weekend: − 17.5 (− 30.9, − 4.1)). All screen behaviours were associated with shorter sleep duration on weekdays, whereas only the use of email/texts and social network sites was associated with shorter sleep duration on weekend days. The association of using social network sites with overall physical activity was stronger in girls than in boys; the association of internet browsing with sedentary behaviour was stronger in boys than in girls.

**Conclusion:**

Intervention strategies to enhance MVPA and sleep duration by limiting screen-based activities may be warranted.

**Supplementary Information:**

The online version contains supplementary material available at 10.1007/s12529-022-10077-7.

## Introduction

Lack of physical activity, excessive screen viewing and inadequate sleep may contribute to an increased risk of the metabolic syndrome, mental health disorders and poor academic attainment in young people [1–6]. Reflecting a growing movement to consider these behaviours holistically, several countries have now issued 24-h movement guidelines for children (5–13 years) and adolescents (14–17 years) [7, 8]. In Canada, for example, young people are recommended to accumulate at least 60 min of moderate-to-vigorous intensity physical activity (MVPA) each day, limit sitting for extended periods with no more than 2 h per day of recreational screen time and attain 8–11 h of sleep each night [9]. Surveillance data indicate that in a 24-h period, children and adolescents in Canada and New Zealand spend approximately half their time sedentary, one-third sleeping and the remainder in light-intensity physical activity and MVPA [10, 11].

The time available each day for physical activity, sedentary behaviour and sleep is finite, such that time spent on one activity has an impact on the availability of time for other activities. The displacement hypothesis asserts that time spent in one behaviour (e.g. sitting) displaces that in another (e.g. physical activity) [12], although the evidence to support this hypothesis appears inconsistent. Review evidence indicates that some types of sedentary behaviour may be negatively associated with physical activity [13], but the size of the association is small, suggesting that these behaviours do not directly displace one another. However, much of the previous work on this topic has focussed on traditional forms of screen use, such as playing video games or watching broadcast television on a television set, failing to account for new devices or modes of screen-based entertainment that have emerged in recent years. This is limiting given that in 2019 approximately 70% of youth aged 12–15 years had a social media account in the UK [14] and spent approximately 3 h per day on these services [15]. Recent evidence indicates that smartphone and tablet use may be negatively associated with self-reported physical activity, though the strength of this association may vary with age and sex [16, 17]. Similarly, previous studies have found that screen time (mainly television viewing and video games) [18, 19] and engagement in social media use (social networking or messaging sites or Apps on the internet) [20] are associated with late sleep onset. Nevertheless, there remains limited evidence of how contemporary screen behaviours (such as time spent in social networking sites and email/texts) may impact on overall sedentary time, or on time spent active or sleeping. A clearer understanding of how these behaviours interact may help to inform the content of behaviour change interventions.

Inconsistency of the evidence regarding displacement between health behaviours may, in part, be attributable to use of different methods to assess these behaviours, which may have varied by behaviour sub-type, recall period or temporal unit [21]. This is in addition to known limitations of self-report behaviour questionnaires, such as recall bias [22]. An alternative to questionnaires for the assessment of specific behaviours is a time-use diary, which have been used to describe patterns of physical activity and sedentary behaviour in young people. Although numerous studies have deployed time-use diaries to assess sedentary and active behaviours in young people, much of this previous research has looked at a limited range of behaviours or used composite markers [23–25], which might mask associations between individual behaviours.

The aim of this study is to examine the association of diary-assessed screen behaviours with overall physical activity, MVPA, sedentary behaviour and sleep in adolescents and explore whether these associations vary by sex. We hypothesised that screen behaviours would be associated with a lower level of physical activity, more time spent sedentary and shorter sleep duration.

## Methods

### Sample and Data Collection

Data are from the Millennium Cohort Study (MCS), a national longitudinal birth cohort study run by the Centre for Longitudinal Studies (CLS) at the University College London. The MCS examines the social, economic, and health-related circumstances of young people born between 2000 and 2002, recruited from all four countries of the UK (England, Scotland, Wales and Northern Ireland) [26, 27]. The MCS is nationally representative and 18,552 families (18,818 children) were recruited at baseline. Seven sweeps of data collection have been undertaken up to 2020, conducted when participants were 9 months and 3, 5, 6, 7, 14 and 17 years of age.

This cross-sectional analysis uses data from the sixth sweep of assessment (MCS6; data collection: January 2015–April 2016), when participants were 14 years old. In MCS6, 15,415 families were contacted for participation; 11,884 participants from 11,726 families provided partial or complete data. A subsample (88%) of young people was invited to wear an activity monitor and complete a time-use diary. The subsample comprised all participants living in Wales, Scotland and Northern Ireland and 81% of participants in England. The English sample was restricted due to limitations on the number of the activity monitors available. The MCS6 was approved by the National Research Ethics Service (NRES) Research Ethics Committee (REC) London – Central (REC ref: 13/LO/1786). Data were anonymised and obtained free of charge from the UK Data Service (http://doi.org/10.5255/UKDA-SN-8156-7). Parents and cohort members provided written and verbal consent prior to completing the survey [28].

### Time-Use Diary

Participants were invited to complete a time-use diary for two randomly chosen days (one weekday and one weekend day) selected by the Computerised Assisted Personal Interviews (CAPI) programme during the interviewer visit. The diary was available in 3 formats: online via the web, App via tablet or phone, and paper. Sixty-four percent of participants selected the App diary format, 29% used the online version and 7% used the paper diary [26]. Participants recorded their behaviour in 10-min timeslots from 4 to 4 am the next day. For each 10-min timeslot, participants indicated their main activity, selecting from a pre-specified list of 44 activities, nested within 12 categories (the full list of activity codes is presented in Electronic Supplementary Material: Table 1). Diaries (days) with missing data (one or more timeslots with no activity indicated) were excluded from the analysis, consistent with previous analyses using time-use diary data [29].

#### Screen Behaviour

Participants were categorised according to whether they did (user) or did not (non-user) report time in the following four activities: phone calls, email and texting, using social network sites and internet browsing. Preliminary analyses indicated that the duration of time spent in these individual behaviours was low and highly skewed; therefore, we opted to dichotomise in all analyses focusing on individual behaviours. We derived a summary duration variable, calculated as the sum of time spent in the 4 activities of interest.

#### Sedentary Behaviour

We derived an outcome variable to indicate time spent in other sedentary behaviours by summing time reported in the following activities: reading for school or pleasure, traveling by car/bus, playing electronic games and TV viewing.

### Accelerometer Data

#### Physical Activity

To provide an assessment of physical activity, participants wore a triaxial GENEActiv Original accelerometer [30] (Activinsights Ltd, Kimbolton, UK) on the non-dominant wrist for the same days as time-use diaries were completed. Data were downloaded using GENEActiv software and raw data processed using the GGIR package in R, which includes autocalibration and non-wear detection functions [31]. Data were collected in 5-s epochs and the analysis includes all days with 10 or more valid hours (i.e. a valid day was defined as one in which wear time exceeded 10 h). Overall physical activity was estimated using the Euclidean Norm Minus One (ENMO), a measure of mean acceleration over a 24-h period. Duration of MVPA was calculated as the time spent with ENMO ≥ 100 mg [32].

### Self-Reported Data

#### Sleep Duration

Participants self-reported their usual time of sleep onset and waking up, separately for week and weekend days, selecting from pre-defined response categories (Electronic Supplementary Material Table 2). Sleep duration was estimated as the time elapsed between category mid-points for sleep onset and wake time, consistent with previous research [33]. Sleep duration estimates were collapsed into four categories (≤ 7 h, 7–8 h, 8–9 h, > 9 h) for weekday sleep duration and three categories (7–8 h, 8–9 h, > 9 h) for weekend sleep duration.

### Covariates

Participants sex, family income, ethnicity, body mass index (BMI) and home location (rural or urban classification) were included as potential covariates in the analysis [34]. Inclusion of covariates in the model was based on previous research that showed association of sex, family income, ethnicity, BMI, home location with screen behaviours and physical activity and sleep. Adjustment for these variables is also consistent with previous research that has examined associations between similar exposures and outcomes as the current study [35, 36]. Rural or urban home location, based on postal code, was derived on the basis of population density [37]. Family income was measured using the Organisation for Economic Co-operation and Development (OECD) equivalised income quintiles, based on parent-reported household income. Ethnicity was parent-reported and categorised as White, Mixed, Indian, Pakistani and Bangladeshi, Black or Black British, and Other ethnic group (including Chinese). Weight and height were measured by trained research assistants. Body mass index (BMI) was calculated as weight divided by height squared (kg/m^2^) and International Obesity Task Force (IOTF) thresholds were used to categorise participants as underweight/normal weight, overweight and obese [38].

### Statistical Analysis

Analyses were conducted in STATA 16.0 (Stata Corporation, TX, USA). Sample characteristics and daily duration of exposure and outcome variables were summarised using descriptive statistics. Sex differences in duration of exposure and outcome variables were examined using the Mann–Whitney *U* tests, Student’s *t*-tests and chi-square tests for continuous and categorical variables. Baseline characteristics for those included and lost to follow-up were compared using Student’s *t*-tests and chi-square tests. Multiple linear regression models were used to examine the association between exposure variables and physical activity outcome variables, separately for weekdays and weekend days. Ordinal logistic regression models were used to examine the association between exposure variables and sleep duration categories. Proportional odds ratios from these models indicate the effect of a 1-unit increase in the exposure on the odds of having longer sleep duration relative to all combined shorter sleep durations, controlling for other variables in the model. The Brant test was used to test for violations of the proportional odds assumption. The association between exposure variables and sedentary behaviour was examined using hurdle models [39], to account for the large number of zero values observed in the sedentary behaviour outcome. The Hurdle model has two parts: [1] a probit component where the outcome is dichotomised (no sedentary time vs. any sedentary time) and (2) a linear regression component which models duration of time spent in sedentary behaviour for non-zero values. We report the linear component in this paper, using the delta method (margins effect) to estimate the mean difference in the duration of sedentary behaviour in those who did/did not report the screen behaviours of interest. For our composite screen behaviour exposure variable, we present the estimated mean difference in sedentary behaviour for a 10-min increase in screen time. All models were adjusted for sex, BMI category, ethnicity, family income and home location. Assumptions of the fitted models were explored with tests for normality, checking for homoscedasticity and collinearity. In all cases, assumptions were not violated. Possible multicollinearity in regression analysis was explored with the variance inflation factor (VIF). In all cases, VIFs were ≤ 2, indicating minimal collinearity amongst variables in the model. Single screen behaviours were modelled simultaneously (mutually adjusted). The composite screen behaviour exposure was modelled separately. Interaction terms were added to regression models to examine effect modification by sex.

## Results

Data from 8,625 diaries were available, of which 1,537 were excluded due to missing data. The analytical samples for weekday and weekend analyses were *n* = 3595 and *n* = 3580 respectively. Table 1 describes the characteristics of participants for the weekday sample. There were no differences in participant characteristics between the weekday and weekend samples. Participants were 14.2 (0.3) years of age, mainly of White ethnicity (85%), normal weight (76%) and mostly living in urban areas (74%). Participants included in the analyses were more likely to be of White ethnicity (*P* < 0.001), have normal weight (*P* < 0.05) and come from families with higher income (*P* < 0.05) compared to those who were excluded.

### Associations between Screen-Based Behaviours and Physical Activity

Cross-sectional associations between screen-based behaviours and physical activity are presented in Table 4. We found no association between making phone calls or sending emails/texts and either of the physical activity outcomes. Use of social network sites was associated with lower overall physical activity on weekend days and fewer minutes of MVPA on weekdays and weekend days. Internet browsing was associated with lower physical activity and MVPA on both weekdays and weekend days. A 10-min increase in duration of screen behaviours was associated with lower physical activity and MVPA on both weekdays and weekend days. Tests for interaction by sex revealed that associations between the use of social network sites, email/text and physical activity and MVPA on weekends were stronger in girls than in boys (Electronic Supplementary Material, Table 4 and 5). For example, compared to non-users, use of social networking sites was not associated with MVPA in boys (− 3.4 (− 12.3, 5.4)) but negatively associated in girls (− 15.3 (− 22.3, − 8.40); *P* for interaction < 0.05).

### Associations between Screen-Based Behaviours and Sedentary Behaviour

Transformed hurdle model outputs indicating the association of screen-based behaviours with sedentary behaviours are presented in Table 5. Untransformed coefficients from the hurdle model are provided in Electronic Supplementary Material, Table 3. The use of social network sites was associated with approximately 19 and 17 fewer minutes of sedentary behaviour on both weekdays and weekends respectively. A 10-min increase in the duration of screen-based behaviours was associated with 3 and 4 fewer minutes in sedentary behaviour on both weekdays and weekends respectively. Tests for interaction by sex revealed that the use of internet browsing on weekends was negatively associated with sedentary behaviour in boys (− 57.7 (− 89.0, − 26.4)) but was not associated in girls (18.8 (− 12.7, 50.4); *P* for interaction < 0.05) (Electronic Supplementary Material, Table 6).

### Associations between Screen-Based Behaviours and Sleep

Associations between screen-based behaviours and sleep duration are presented in Table 6. Participants who reported making phone calls or browsing the internet were less likely to attain ≥ 9 h of sleep on weekdays. Adolescents using email/text and social network sites were less likely to attain ≥ 9 h of sleep on both weekdays and weekend days. A 10-min increase in the duration of screen-based behaviours was associated with lower odds of attaining ≥ 9 h of sleep on both weekdays and weekend days. Test for interactions by sex showed that making phone calls was associated with lower odds of ≥ 9 h of sleep on weekends in girls (0.62 (0.41, 0.93)) but was not associated with sleep duration in boys (1.41 (0.77, 2.59); *P* for interaction < 0.05) (Electronic Supplementary Material, Table 7).

## Discussion

This study examined the association of selected screen-based behaviours with physical activity, sedentary behaviour and sleep in adolescents and explored whether the association varied by sex. The results show that participation in some screen-based behaviours and the duration of all screen-based behaviours are associated with less overall physical activity and MVPA, less sedentary behaviour and shorter sleep duration on both weekdays and weekend days. A small number of differences in the direction or magnitude of these associations was observed between boys and girls, which may have implications for intervention design.

The use of social network sites and internet browsing was associated with lower overall PA and 5 to 10 fewer minutes of MVPA on both weekdays and weekend days. Our findings are consistent with previous evidence [17, 40], which showed that time spent in contemporary screen-based behaviours (i.e. tablet, smartphone and social media) was associated with insufficient levels of PA (PA < 60 min), measured by self-report questionnaire. However, our findings contrast with those from a previous study in Norwegian adolescents, which reported that socialising and surfing online were not associated with physical activity [41]. These contrasting results may be due to geographic variability in how these behaviours interact. In a cross-national investigation [42], strong negative associations between physical activity and screen-based sedentary behaviours were found in North America and the Nordic countries, but associations were generally weaker in the British Isles, Central Europe and the Baltic countries. Few studies to date have examined the association between screen behaviours and vigorous intensity physical activity; this would be a valuable avenue for future research given the known health benefits of vigorous intensity physical activity. Our findings indicate a complex suite of associations between screen-based activities and adolescents’ physical activity, which may vary by behaviour and location amongst other things. Negative associations of visiting social networking sites and internet browsing with physical activity provide partial support for the displacement hypothesis, but the associations were generally small in magnitude, consistent with review evidence [43, 44], particularly when considering the duration of use rather than doing/not doing these behaviours. Nonetheless, strategies to reduce time spent in specific screen behaviours may be valuable as part of a package of measures in programmes aiming to promote physical activity in adolescents.

Surprisingly, the use of social network sites and the duration of screen-based behaviours were associated with less composite sedentary behaviour on both weekdays and weekend days. The scarcity of evidence on the associations of contemporary screen time with sedentary behaviour makes the comparison of our findings with prior research difficult. However, a previous study showed that the presence of TV in the bedroom and combined presence of computer and TV set were negatively associated with accelerometer-assessed sedentary time [45]. There are several possible explanations for these findings. Firstly, adolescents may spend time using social media via portable devices, such as mobile phones, whilst engaging in light activity, and are not necessarily sedentary. A study using data from two UK time-use surveys (2000–2015) found an increase in the time children spent using mobile devices and tablets when engaging in other activities throughout the day (i.e. time at school, during travel, and when eating) [46]. Research to establish body posture or the presence/absence of activity whilst using screen-based devices will advance our understanding on how screen behaviours may displace time in sedentary behaviour. Another potential explanation is that the negative associations of screen behaviours with sedentary behaviour may be due to the changes in media use and the shift from traditional (e.g. TV viewing, video games) to contemporary screen use behaviours in the current generation. Our composite measure of sedentary behaviour consists of the sum of screen- and non-screen-based sedentary activities; therefore, it may be hypothesised that more time in social networking sites was associated with less TV viewing, video game playing or reading for school or leisure, all of which are predominantly sedentary activities.

We found that all four of the screen-based behaviours examined were associated with shorter sleep duration on weekdays, and the use of email/texts and social network sites was associated with shorter sleep on weekend days. Our findings add to a growing body of evidence indicating that the use of screen devices (both traditional and contemporary) is associated with shorter sleep duration (i.e. less than 8 h) in this population [17, 47–49]. However, much of the previous research has examined whole week patterns in sleep behaviour, without distinguishing week and weekend days. This knowledge can help with the targeting and content of behaviour change interventions. Differences in the association observed across week and weekend days may reflect the differing daily routines of young people during the week/weekend, and the differing times of day when adolescents can engage in these activities. However, it would be valuable to see if these differences were replicated in further analyses before drawing firm conclusions. Given that short and interrupted sleep may have implications for adolescents’ mental health and well-being [49–51], these findings support the development of strategies to monitor screen time in programmes aimed at promoting healthy sleep habits in adolescents. Further research to corroborate our findings, however, should be undertaken prior to application of these strategies in practice.

This is one of the few existing studies that has examined whether associations of screen behaviours with physical activity, sedentary behaviour and sleep vary by sex. A number of significant interactions were observed, sometimes in opposing directions. For example, use of social networking sites was associated with 15 fewer minutes of MVPA in girls, but not in boys. This is consistent with prior evidence showing that the use of social media and chat apps for four or more hours per day was negatively associated with MVPA in girls, but no such association was observed in boys [52]. We also found that the association between internet browsing and sedentary behaviour was stronger in boys but not in girls. However, this finding is not consistent with evidence on bedroom media which showed that the negative association of television and computer ownership with sedentary time was stronger in girls than in boys [45]. Evidence on variations in the associations between screen behaviours and movement behaviours by sex is inconsistent at this point. In addition, few studies have formally tested for effect modification by sex. Further studies are required to examine whether the associations between screen behaviours, physical activity, sedentary behaviour and sleep vary by sex. This will help to inform the content and targeting of behaviour change interventions addressing this suit of health-related behaviours.

Our findings cannot be used to determine causality, due to the cross-sectional design, but they do nonetheless add to the evidence base concerning inter-relations between health behaviours, particularly given our focus on contemporary screen behaviours, which have been little studied in this context to date. Previous research suggested that there is time for both screen activities (traditional devices) and physical activity and therefore provided limited support for the displacement hypothesis [53]. Additionally, our findings indicate differential associations between specific screen activities and other health behaviours; use of social network sites was consistently associated with adolescents’ physical activity, sedentary behaviour and sleep duration for example, whilst making phone calls or using email/texting was associated with sleep only. These nuances further our understanding of the complex pathways that link behaviour with health and can guide the development of behaviour change interventions. Where appropriate, advanced analytical techniques, such as compositional analysis, can further our understanding of how particular behaviours, or groups of behaviours, interact within our daily time budget [54].

### Strengths and Limitations

A strength of this study is the large geographically and demographically diverse sample. In addition, we utilised device-based measures of overall PA and MVPA, reducing the bias associated with self-report. Regression models included adjustments for known confounders, and we explored effect modification by sex. Lastly, the use of time-use diary-derived data allowed us to study contemporary screen behaviours, such as use of social networking sites, which have been relatively understudied in this field to date. Nevertheless, our results should be interpreted with the following limitations in mind. Firstly, the results are derived from a British population and, as such, conclusions may not be fully generalisable to other nations. Secondly, due to the cross-sectional nature of the analysis, we cannot determine the direction of the associations observed. Thirdly, the time-use diaries did not provide information on the type of device (e.g. tablet or smartphone, portable or non-portable) used whilst reporting time in screen behaviour which may have introduced variability into the associations of interest and limits direct applicability to the development of intervention strategies. Fourthly, we acknowledge that a substantial number of participants were excluded from the analysis due to missing diary data, consistent with previous research using this methodology. Our analytical sample differed in a number of social and demographic characteristics to the wider cohort, potentially limiting the generalisability of our findings. Lastly, the validity of the specific time-use diary used in this study is unknown, though it was rigorously pilot-tested prior to use and diaries of a similar nature have demonstrated acceptable validity and reliability [55].

## Conclusions

In this study, the use of social network sites and internet browsing were consistently associated with less MVPA and sedentary behaviour on both weekdays and weekend days, and the use of all screen behaviours was strongly associated with shorter sleep duration on weekdays. In light of continued growth in ownership and usage of screen-based devices in young people, further work to understand how these activities interact with other behaviours, including physical activity and sleep, is warranted. Our findings indicate that intervention strategies to limit screen behaviours may be valuable components in programmes aimed at promoting MVPA and adequate sleep in this age group, along with appropriate tailoring by sex in some instances.

**Table 1:** Participant characteristics (weekday sample)

	**All (*n* = 3595)**	**Boys (*n* = 1612)**	**Girls (*n* = 1983)**
**Age, mean ± SD, y**	14.2 (0.3)	14.2 (0.3)	14.2 (0.3)
**Ethnicity, *n* (%)**			
White	3043 (85)	1351 (84)	1692 (85)
Mixed	142 (4)	79 (4)	63 (3)
Indian	94 (3)	47 (2)	47 (2)
Pakistani and Bangladeshi	170 (4)	69 (4)	101 (5)
Black or Black British	61 (1)	31 (1)	30 (1)
Other ethnic group	67 (1)	24 (1)	43 (2)
**Family income (quintile, *n* (%))**			
First (lowest)	338 (9)	124 (7)	214 (10)
Second	450 (12)	194 (12)	256 (12)
Third	710 (19)	319 (19)	391 (19)
Fourth	994 (27)	458 (28)	536 (27)
Fifth (highest)	1100 (30)	515 (31)	585 (29)
**BMI (IOTF classification), *n* (%)**			
Normal weight (incl. underweight)	2685 (76)	1250 (78)	1435 (75)
Overweight	606 (17)	257 (16)	349 (18)
Obese	216 (6)	92 (5)	124 (6)
**Home location, *n* (%)**			
Rural	908 (25)	391 (24)	517 (26)
Urban	2681 (74)	1217 (75)	1464 (73)

*SD* standard deviation, *y* year

Sample sizes vary due to missing data: Ethnicity: All = 3577, B = 1601, G = 1976; Family income: All = 3592, B = 1610, G = 1982; BMI (International Obesity Task Force (IOTF)): All = 3507, B = 1599, G = 1908; Home location: All = 3589, B = 1608, G = 1981

Usage and duration of selected screen-based behaviours are presented in Table 2. The proportion of participants that reported usage of phone calls, email/text, and internet browsing was less than 20% during the week and at the weekend, with boys being less likely to report doing these activities than girls. Approximately 40% of participants reported time spent on social network sites. This was more likely on the weekend than during the week, and in girls than in boys. Time spent on the 4 screen behaviours combined was greater at the weekend than during the week (median (IQR) 30 min (0, 90) vs. 20 min (0, 80)). Time estimates for MVPA, sedentary behaviour and sleep are presented in Table 3

**Table 2:** Number and proportion of participants reporting use of selected screen-based behaviours and duration of summed screen behaviours (values are *N* (%) unless stated otherwise)

**Exposure variables**	**Weekday**				**Weekend**			
	**Users**		**Non-users**		**Users**		**Non-users**	
	**Boys**	**Girls**	**Boys**	**Girls**	**Boys**	**Girls**	**Boys**	**Girls**
Phone calls	75 (4.6)	159 (8)	1537 (95.3)	1824 (91.9)*	95 (5.9)	199 (10)	1504 (94.0)	1782 (89.9)*
Email/text	180 (11.1)	370 (18.6)	1432 (88.8)	1613 (81.3)*	204 (12.7)	380 (19.1)	1395 (87.2)	1601 (80.8)*
Social network sites	421 (26.1)	974 (49.1)	1191 (73.8)	1009 (50.8)*	406 (25.3)	1042 (52.6)	1193 (74.6)	939 (47.4)*
Internet browsing	251 (15.5)	269 (13.5)	1361 (84.4)	1714 (86.4)	260 (16.2)	302 (15.2)	1339 (83.7)	1679 (84.7)
Screen behaviour (min), median (IQR)	**All**	**Boys**	**Girls**		**All**	**Boys**	**Girls**	
	20 (0, 80)	0 (0, 60)	30 (0, 90)*		30 (0, 90)	0 (0, 60)	40 (0, 120)*	

*IQR* inter-quartile range

Screen behaviour weekday sample: All = 3595, B = 1612, G = 1983. Screen behaviour weekend sample: All = 3580, B = 1599, G = 1981

*Differences between sex (*P* value < 0.001)

**Table 3:** Duration of overall and moderate-to-vigorous physical activity, sedentary behaviour and sleep

**Outcome variables**	**Weekday**			**Weekend**		
	**All**	**Boys**	**Girls**	**All**	**Boys**	**Girls**
MVPA (min), mean ± SD	135.6 (62.7)	143.1 (67.4)	128.7 (57.1)*	114.3 (64.9)	117.8 (70.6)	111.2 (59)*
Overall physical activity (mean acceleration; ENMO), mean ± SD	35.2 (15.4)	38.4 (17.6)	32.2 (12.2)*	31.2 (15.5)	33.6 (18)	29.1 (12.4)*
Composite of sedentary behaviour (min), median (IQR)	200 (110, 310)	240 (120, 360)	180 (100, 270)*	270 (150, 410)	330 (180, 470)	240 (120, 350)*
Self-reported sleep duration, *n* (%)						
≤ 7 h	1359 (11.8)	599 (10.4)	760 (13.1)*	0	0	0
7–8 h	3375 (29.3)	1600 (28.0)	1775 (30.7)	233 (20)	121 (2.1)	112 (2.9)*
8–9 h	4870 (42.4)	2438 (42.6)	2432 (42.1)	1545 (13.4)	874 (15.3)	671 (11.6)
> 9 h	1882 (16.3)	1076 (18.8)	806 (13.9)	9708 (84.5)	4718 (82.5)	4990 (86.4)

*IQR* inter-quartile range, *SD* standard deviation, *ENMO* Euclidean Norm Minus One, *min* minutes, *hrs* hours

Accelerometer variables for MVPA and overall physical activity: weekday sample: All = 4546, B = 2196, G = 2350; weekend sample: All = 4457, B = 2127, G = 2330. Composite of sedentary behaviour variable: weekday sample: All = 3551, B = 1596, G = 1955; weekend sample: All = 3537, B = 1582, G = 1955

*Differences between sex (*P* value < 0.001)

**Table 4:** Cross-sectional association between screen-based behaviours and accelerometer-assessed overall and moderate-to-vigorous physical activity

	**Overall physical activity**			
	**Weekday**		**Weekend**	
	***β* (95% CI)**	***P* value**	***β* (95% CI)**	***P* value**
Phone calls	− 1.5 (− 3.8, 0.82)	0.20	− 0.18 (− 2.40, 2.04)	0.87
Email/text	0.42 (− 1.2, 2.07)	0.61	− 0.95 (− 2.64, 0.73)	0.26
Social network sites	− 1.0 (− 2.30, 0.20)	0.10	− 1.9 (− 3.25, − 0.60)	0.004
Internet browsing	− 2.6 (− 4.28, − 0.92)	0.002	− 2.48 (− 4.15, − 0.80)	0.004
Screen behaviour^≠^	− 0.21 (− 0.27, − 0.14)	< 0.001	− 0.20 (− 0.26, − 0.14)	< 0.001
	**Moderate-to-vigorous physical activity**			
	**Weekday**		**Weekend**	
	***β* (95% CI)**	***P* value**	***β* (95% CI)**	***P* value**
Phone calls	− 5.31 (− 14.9, 4.31)	0.27	− 1.67 (− 10.9, 7.57)	0.72
Email/text	1.73 (− 5.03, 8.51)	0.65	− 3.54 (− 10.5, 3.4)	0.32
Social network sites	− 5.21 (− 10.3, − 0.04)	0.04	− 10.0 (− 15.5, − 4.5)	< 0.001
Internet browsing	− 10.6 (− 17.5, − 3.69)	0.003	− 10.8 (− 17.8, − 3.8)	0.002
Screen behaviour^≠^	− 0.88 (− 1.16, − 0.60)	< 0.001	− 0.90 (− 1.16, − 0.65)	< 0.001

^≠^A change in outcome variable (min/day) for 10-min increase in screen behaviour

Phone calls, Email/text, Social network sites, Internet browsing: reference group is non-users

*MVPA* moderate-to-vigorous intensity physical activity, *β* beta coefficient, *95% CI* 95% confidence interval

**Table 5:** Cross-sectional association between screen-based behaviours and composite sedentary behaviours

	**Composite sedentary behaviour**			
	**Weekday**		**Weekend**	
	**Dy/dx (95% CI)**	***P* value**	**Dy/dx (95% CI)**	***P* value**
Phone calls	− 13.6 (− 34.8, 7.6)	0.21	− 1.5 (− 25.2, 22.1)	0.89
Email/text	− 9.3 (− 24.2, 5.6)	0.22	− 17.5 (− 35, − 0.0)	0.04
Social network sites	− 19.8 (− 31, − 8.6)	< 0.001	− 17.5 (− 30.9, − 4.1)	0.01
Internet browsing	− 0.7 (− 16.3, 14.7)	0.92	6.1 (− 11.6, 24)	0.49
Screen behaviour^≠^	− 3.6 (− 4.3, − 2.9)	< 0.001	− 4.3 (− 5, − 3.6)	< 0.001

^≠^A change in outcome variable (min/day) for 10-min increase in screen behaviour

Phone calls, Email/text, Social network sites, Internet browsing: reference group is non-users

*Dy/dx* average marginal effect of dx (screen behaviours) on dy (sedentary behaviour), *95% CI* 95% confidence interval

**Table 6:** Cross-sectional association between screen-based behaviour and sleep duration

	**Sleep duration**			
	**Weekday**		**Weekend day**	
	**POR (95% CI)**	***P* value**	**POR (95% CI)**	***P* value**
Phone calls	0.78 (0.61, 1.0)	0.05	0.86 (0.61, 1.22)	0.41
Email/text	0.80 (0.67, 0.95)	0.01	0.76 (0.59, 0.99)	0.04
Social network sites	0.78 (0.68, 0.89)	< 0.001	0.78 (0.61, 1.00)	0.05
Internet browsing	0.75 (0.62, 0.89)	0.002	1.07 (0.87, 1.32)	0.47
Screen behaviour^≠^	0.96 (0.95, 0.96)	< 0.001	0.98 (0.97, 0.99)	< 0.001

^≠^A change in outcome variable (odd ratio/day) for 10-min increase in screen behaviour

Phone calls, Email/text, Social network sites, Internet browsing: reference group is non-users

*POR*, proportional odd ratio; *95% CI*, 95% confidence interval

## Supplementary Information

Below is the link to the electronic supplementary material.

Supplementary file1 (DOCX 72 KB)
