# Supplementary material for: The Association of Contemporary Screen Behaviours with Physical Activity, Sedentary Behaviour and Sleep in Adolescents: a Cross-sectional Analysis of the Millennium Cohort Study
Source: Int J Behav Med. 2022 Mar 11;30(1):122–32. doi: 10.1007/s12529-022-10077-7 (PMC9879798; doi:10.1007/s12529-022-10077-7)
Supplement: Supplementary file 1 — Supplementary file1 (DOCX 72 KB) [file 12529_2022_10077_MOESM1_ESM.docx]

Title: Title: The association of contemporary screen-behaviours with physical activity, sedentary behaviour and sleep in adolescents: a cross-sectional analysis of the Millennium Cohort Study.

| Categories | Codes |
| --- | --- |
| Sleep and personal care | Sleeping and resting; Personal care |
| School, homework, and education | Homework; In class; School breaks; School clubs; Detention |
| Paid or unpaid work | Paid work; Unpaid work for family or other non-household members |
| Chores, housework, and looking after people or animals | Cooking, cleaning, and shopping for the household; Fixing things around the house, fixing bike, gardening; Looking after siblings in the household; Looking after parent or other adult in the household (medical or personal care); Looking after animals |
| Eating and drinking | Eating or drinking in a restaurant or café; Eating a meal; Eating a snack or having a drink |
| Physical exercise and sports | Cycling; Ball games and training; Jogging, running, walking, hiking; Team ball games and training; Swimming and other water sports; Other physical exercise and other sports |
| Travelling (including walking to school) | Travel by bus, taxi, tube, plane; Travel by car, van (including vehicles owned by friends and family); Travel by physically active means (walk, bike etc.) |
| Social time and family time | Attending live sporting events; Cinema, theatre, performance, gig; Exhibition, museum, library, other cultural events; Shopping; Speaking on the phone; Speaking, socialising face-to-face |
| Internet, TV, and digital media | Answering emails, instant messaging, texting;  Browsing and updating social networking sites (e.g. Twitter, Facebook, BBM, Snapchat);  General internet browsing, programming (not time on social networking sites); Listening to music, radio, iPod, other audio content;  Playing electronic games and Apps; Watching TV, DVDs, downloaded videos |
| Volunteering and religious activities | Volunteering; Religious activities |
| Hobbies and other free time activities | Did nothing, just relaxing, bored, waiting; Hobbies, arts and crafts, musical activities, writing stories, poetry; Reading (not for school) |
| Any other activity | Other activities not listed |

# **Electronic Supplementary Material**

**Table 1.** Full list of categories and codes (behaviours) for time-use diary.

**Table 2.** Self-completed questions on sleep habits.

| About what time do you usually go to sleep on a school night? |
| --- |
|  |
| 1. Before 9 pm |
| 2. 9 - 9:59 pm |
| 3. 10 – 10:59 pm |
| 4. 11 - midnight |
| 5. After midnight |
|  |
| About what time do you usually wake up in the morning on a school day? |
|  |
| 1. Before 6 am |
| 2. 6 - 6:59 am |
| 3. 7 – 7:59 am |
| 4. 8 - 8:59 am |
| 5. After 9 am |
|  |
| About what time do you usually go to sleep on the nights when you do not have school the next day? |
|  |
| 1. Before 9 pm |
| 2. 9 - 9:59 pm |
| 3. 10 - 10:59 pm |
| 4. 11 - midnight |
| 5. After midnight |
|  |
| About what time do you wake up in the morning on the days when you do not have school? |
|  |
| 1. Before 8 am |
| 2. 8 - 8:59 am |
| 3. 9 – 9:59 am |
| 4. 10 - 10:59 am |
| 5. 11 - 11:59 am |
| 6. After Midday |

**Table 3.** Hurdle model: Linear regression for the association of screen-based behaviour with sedentary behaviour.

|  | **Composite sedentary behaviour** | | | |
| --- | --- | --- | --- | --- |
|  | **Weekday** | | **Weekend** | |
|  | **β (95% CI)** | ***P* value** | **β (95% CI)** | ***P* value** |
| Phone calls | -.17 (-.41, .06) | 0.14 | 0.29 (0.00, 0.57) | 0.04 |
| Email/text | 0.12 (-.06, 0.31) | 0.21 | 0.05 (-0.13, 0.24) | 0.59 |
| Social network sites | 0.31 (0.16, 0.45) | <0.001 | 0.29 (0.14, 0.44) | <0.001 |
| Internet browsing | 0.42 (0.19, 0.66) | <0.001 | 0.40 (0.16, 0.63) | <0.001 |
| Screen behaviour | -.01 (-0.01, -0.00) | <0.001 | -.00 (-.01, -.00) | <0.001 |

Β, beta coefficient; 95% CI, 95% Confidence Interval.

**Table 4**. Interaction by sex for the association between screen-based behaviour and overall physical activity.

|  | **Overall physical activity** | | | |
| --- | --- | --- | --- | --- |
|  | **Weekday** | | **Weekend** | |
|  | **β (95% CI)** | ***P* value** | **β (95% CI)** | ***P* value** |
| Phone calls |  |  |  |  |
| Boys | -0.74 (-5.07, 3.58) | 0.73 | 0.22 (-3.51, 3.97) | 0.59 |
| Girls | -2.09 (-4.86, 0.67) | 0.13 | -1.03 (-3.78, 1.72) | 0.46 |
| Sex*phone calls | -1.35 (-6.48, 3.78) | 0.60 | -1.25 (-5.90, 3.38) | 0.59 |
| Email/text |  |  |  |  |
| Boys | -0.16 (-2.91, 2.59) | 0.90 | 0.47 (-2.29, 3.23) | 0.73 |
| Girls | 0.26 (-1.77, 2.30) | 0.79 | -2.53 (-4.63, -0.43) | 0.01 |
| Sex*Email/text | 0.42 (-3.00, 3.85) | 0.80 | -3.00 (-6.48, 0.46) | 0.09 |
| Social network sites |  |  |  |  |
| Boys | -0.58 (-2.60, 1.42) | 0.56 | -0.29 (-2.42, 1.82) | 0.78 |
| Girls | -1.65 (-3.24, -0.06) | 0.04 | -3.23 (-4.90, -1.55) | 0.001 |
| Sex* Social network sites | -1.06 (-3.63, 1.49) | 0.41 | **-**2.93 (-5.63, -0.22) | **0.03** |
| Internet browsing |  |  |  |  |
| Boys | -4.18 (-6.56, -1.80) | 0.001 | -1.68 (-4.16, 0.79) | 0.18 |
| Girls | -1.29 (-3.63, 1.05) | 0.27 | -3.52 (-5.77, -1.27) | 0.002 |
| Sex* Internet browsing | 2.89 (-0.45, 6.23) | 0.09 | -1.83 (-5.18, 1.50) | 0.28 |
| Screen behaviour |  |  |  |  |
| Boys | -0.23 (-0.33, -0.13) | 0.001 | -0.16 (-0.25, -0.07) | 0.001 |
| Girls | -0.19 (-0.26, -0.12) | 0.001 | -0.20 (-0.27, -0.14) | 0.001 |
| Sex* Screen behaviour | 0.04 (-0.09, 0.18) | 0.52 | -0.06 (-0.18, 0.05) | 0.30 |

Β, beta coefficient; 95% CI, 95% Confidence Interval.

**Table 5.** Interaction by sex for the association between screen-based behaviour and MVPA.

|  | **Moderate-to-vigorous physical activity** | | | |
| --- | --- | --- | --- | --- |
|  | **Weekday** | | **Weekend** | |
|  | **β (95% CI)** | ***P* value** | **β (95% CI)** | ***P* value** |
| Phone calls |  |  |  |  |
| Boys | -1.65 (-19.50, 16.20) | 0.85 | -1.19 (-16.81, 14.42) | 0.88 |
| Girls | -8.11 (-19.5, 3.30) | 0.16 | -4.84 (-16.3, 6.64) | 0.40 |
| Sex*phone calls | -6.46 (-27.66, 14.72) | 0.55 | -3.65 (-23.03, 15.72) | 0.71 |
| Email/text |  |  |  |  |
| Boys | -1.88 (-13.2, 9.47) | 0.74 | 3.34 (-8.20, 14.8) | 0.57 |
| Girls | 1.66 (-6.75, 10.0) | 0.69 | -11.2 (-20.0, -2.49) | 0.01 |
| Sex*Email/text | 3.55 (-10.5, 17.7) | 0.62 | -14.6 (-29.1, -0.10) | **0.04** |
| Social network sites |  |  |  |  |
| Boys | -2.91 (-11.2, 5.3) | 0.49 | -3.45 (-12.3, 5.41) | 0.44 |
| Girls | -7.90 (-14.4, -1.35) | 0.01 | -15.3 (-22.3, -8.40) | 0.001 |
| Sex* Social network sites | -4.98 (-15.5, 5.59) | 0.35 | -11.9 (-23.2, -0.65) | **0.03** |
| Internet browsing |  |  |  |  |
| Boys | -13.7 (-23.6, -3.94) | 0.006 | -7.14 (-17.4, 3.19) | 0.17 |
| Girls | -8.62 (-18.3, 1.04) | 0.08 | -15.7 (-25.1, -6.37) | 0.001 |
| Sex* Internet browsing | 5.16 (-8.63, 18.9) | 0.46 | -8.60 (-22.5, 5.34) | 0.22 |
| Screen behaviour |  |  |  |  |
| Boys | -0.93 (-1.32, -0.51) | 0.001 | -0.69 (-1.07, -0.31) | 0.001 |
| Girls | -0.84 (-1.13, -0.55) | 0.001 | -0.94 (-1.20, -0.67) | 0.001 |
| Sex* Screen behaviour | 0.04 (-0.46, 0.65) | 0.74 | -0.39 (-0.91, 0.12) | 0.13 |

Β, beta coefficient; 95% CI, 95% Confidence Interval.

**Table 6.** Interaction by sex for the association between screen-based behaviour and composite sedentary behaviour.

|  | **Composite sedentary behaviour** | | | |
| --- | --- | --- | --- | --- |
|  | **Weekday** | | **Weekend** | |
|  | **β (95% CI)** | ***P* value** | **β (95% CI)** | ***P* value** |
| Phone calls |  |  |  |  |
| Boys | -0.65 (-54.2, 52.9) | 0.98 | -57.4 (107.2, -7.61) | 0.02 |
| Girls | -36.62 (-80.0, 6.75) | 0.09 | -9.20 (-47.6, 29.2) | 0.63 |
| Sex*phone calls | -35.9 (-104.9, 32.9) | 0.30 | 48.2 (-14.7, 111.1) | 0.13 |
| Email/text |  |  |  |  |
| Boys | -36.0 (-72.5, 0.45) | 0.05 | -63.8 (-99.6, -27.9) | 0.001 |
| Girls | -27.6 (-57.2, 1.89) | 0.06 | -20.3 (-49.8, 9.19) | 0.17 |
| Sex*Email/text | 8.37 (-38.5, 55.3) | 0.72 | 43.4 (-2.91, 89.8) | 0.06 |
| Social network sites |  |  |  |  |
| Boys | -69.8 (-95.5, -44.1) | 0.001 | -57.8 (-84.4, -31.3) | 0.001 |
| Girls | -42.5 (-65.0, -19.9) | 0.001 | -37.8 (-61.0, -14.5) | 0.001 |
| Sex* Social network sites | 27.2 (-6.74, 61.3) | 0.11 | 20.0 (-15.1, 55.2) | 0.26 |
| Internet browsing |  |  |  |  |
| Boys | -50.7 (-81.9, -19.6) | 0.001 | -57.7 (-89.0, -26.4) | 0.001 |
| Girls | -13.5 (-46.7, 19.6) | 0.43 | 18.8 (-12.7, 50.4) | 0.24 |
| Sex* Internet browsing | 37.2 (-8.18, 82.6) | 0.10 | 76.6 (32.1, 121.0) | **0.001** |
| Screen behaviour |  |  |  |  |
| Boys | -6.78 (-8.43, -5.13) | 0.001 | -7.58 (-9.13, -6.03) | 0.001 |
| Girls | -5.5 (-6.47, -4.37) | 0.001 | -5.69 (-6.73, -4.65) | 0.001 |
| Sex* Screen behaviour | -2.80 (-34.4, 28.8) | 0.86 | 1.37 (-30.6, 33.3) | 0.93 |

Β, beta coefficient; 95% CI, 95% Confidence Interval.

**Table 7.** Interaction by sex for the association between screen-based behaviour and sleep duration.

|  | **Sleep duration** | | | |
| --- | --- | --- | --- | --- |
|  | **Weekday** | | **Weekend** | |
|  | **β (95% CI)** | ***P* value** | **β (95% CI)** | ***P* value** |
| Phone calls |  |  |  |  |
| Boys | 0.77 (0.50, 1.17) | 0.23 | 1.41 (0.77, 2.59) | 0.26 |
| Girls | 0.73 (0.54, 1.00) | 0.05 | 0.62 (0.41, 0.93) | 0.02 |
| Sex*phone calls | 0.95 (0.56, 1.61) | 0.86 | 2.27 (1.09, 4.72) | **0.02** |
| Email/text |  |  |  |  |
| Boys | 0.66 (0.49, 0.88) | 0.005 | 0.95 (0.64, 1.40) | 0.80 |
| Girls | 0.82 (0.66, 1.01) | 0.06 | 0.63 (0.45, 0.87) | 0.005 |
| Sex*Email/text | 1.24 (0.86, 1.77) | 0.23 | 1.50 (0.90, 2.50) | 0.11 |
| Social network sites |  |  |  |  |
| Boys | 0.73 (0.59, 0.90) | 0.004 | 0.97 (0.72, 1.31) | 0.89 |
| Girls | 0.75 (0.63, 0.89) | 0.001 | 1.08 (0.81, 1.43) | 0.57 |
| Sex* Social network sites | 1.02 (0.78, 1.33) | 0.85 | 1.10 (0.73, 1.66) | 0.62 |
| Internet browsing |  |  |  |  |
| Boys | 0.63 (0.49, 0.82) | 0.001 | 0.75 (0.53, 1.05) | 0.09 |
| Girls | 0.78 (0.61, 1.00) | 0.05 | 0.77 (0.53, 1.11) | 0.16 |
| Sex* Internet browsing | 1.23 (0.86, 1.74) | 0.24 | 1.02 (0.62, 1.68) | 0.91 |
| Screen behaviour |  |  |  |  |
| Boys | 0.96 (0.95, 0.97) | 0.001 | 0.98 (0.97, 0.99) | 0.04 |
| Girls | 0.96 (0.95, 0.97) | 0.001 | 0.98 (0.97, 0.99) | 0.002 |
| Sex* Screen behaviour | 1.00 (0.98, 1.01) | 0.86 | 0.99 (0.97, 1.01) | 0.51 |

Β, beta coefficient; 95% CI, 95% Confidence Interval.
